# Postoperative meningocele after anterior cervical discectomy and arthroplasty on a case report

**DOI:** 10.11604/pamj.2022.42.257.35812

**Published:** 2022-08-08

**Authors:** Paul Lavantes, Thierry Dufour

**Affiliations:** 1 Institut Parisien du Dos, Clinique Geoffroy-Saint-Hilaire, 59, rue Geoffroy-Saint-Hilaire, 75005 Paris, France

**Keywords:** Cerebrospinal fluid leaked, dural tear, cervical arthroplasty, cervical spine, case report

## Abstract

Anterior dural tears complicated by cerebrospinal fluid (CSF) leakage with anterior meningocele are rare. Indeed, in the literature, cases are described during anterior arthrodesis, but no cases of post-cervical arthroplasty are described. The management of this type of complication is poorly described and not consensual. We present a case of a patient who underwent cervical arthroplasty complicated by an anterior meningocele at 1 month after the first surgery. Imaging revealed a compressive anterior meningocele in relation to the clinically progressive worsening. Revision surgery consisted of a combination of closure of the gap with a fatty patch covered with a TachoSil patch, followed by reinsertion of a new cervical prosthesis. At the last follow-up at 1 year, the patient showed no residual effects of the complication, and the mobility of the disc prosthesis was not impaired by it. Clinical results of the arthroplasty are also very satisfactory. Although these types of complications are rare, it is important to have a consensus on the management of anterior meningocele. In our experience, TachoSil appears to be a satisfactory option for the management of these complications.

## Introduction

Cervical arthroplasty surgery is increasingly used for the management of degenerative cervical disc disease but also for cervical brachial neuralgia and cervico arthrosic myelopathy. This surgery generally has an excellent clinical outcome, and the morbidity and complication rate are generally low. In the literature, the incidence of cerebrospinal fluid leakage following anterior cervical spinal decompression surgery is in the range of 0.5 to 3%. [1]. Most surgeons have very limited experience in the management of these anterior cervical spinal tears, because its rarity. In the cervical spine, the pressure of the CSF is low and one surgical option is to do anything to close these leaks except simply complete the operation and avoid using drainage. Cervical arthroplasty has a good prognosis but may have complications such as postoperative meningocele which is the subject of our case report. In our case, the prosthesis allows communication between the neurological elements and the esophageal and tracheal prevertebral structures. No article in the literature describes the management of an anterior meningocele in the context of cervical arthroplasty.

## Patient and observation

**Patient information:** we present the case of a 48-year-old patient who presented a multilevel degenerative cervical disease with a grade 1 spondylolisthesis in C7-T1disc leading to beginning myelopathy signs (C5-C6, C6-C7 and C7-T1, underwent hybrid surgery, arthroplasties of the C5-C6 and C6-C7 levels (MOBI-C®, Zimmer Biomet) and arthrodesis of the C7-T1 level (ROI-C®, Zimmer Biomet). The initial procedure was performed with the patient in the dorsal position under C-arm fluoroscopy and microscope. During the C5-C6 discectomy, it was noted that a small portion of the posterior longitudinal ligament was adherent to the dura on the right foramina. A punctiform dural tear lateralized to the right side at the level of the right C6 nerve emergence was visualized under the microscope with minimal flow. The placement of a 5 mm TachoSil sponge at the level of the tear allows to dry up the flow immediately. The rest of the surgery was carried out without any complication. The initial postoperative course was simple. The patient was discharged from the department on the first day after surgery, with no postoperative headache and no cervical abnormality, no dysphonia.

**Figure 1:**
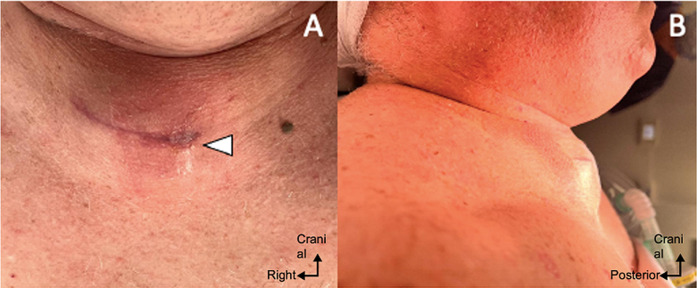
anterior (A) and lateral (B) illustrations (the upper part of the image is the chin) showing a clinical defect at the left part of the scar (white arrow) (A) and a significant compressive curvature (B) in a short neck patient

**Clinical findings:** the patient was seen in consultation at twenty-day post-surgery because of the persistence of a “clear water” flow through a punctiform orifice, laterally to the skin incision, without any clinical or biological signs. He was seen again on 26^th^ day with persistent collection and discharge. No dysphagia or dysphonia was observed, but the leakage was evident. Despite neurological post op improvement from myelopathy signs, it was decided of rehospitalization urgently for 2d surgery with evacuation of the collection, bacteriological sampling and closure of the dural tear at the origin of the meningocele fistulizing to the skin (Figure 1).

**Figure 2:**
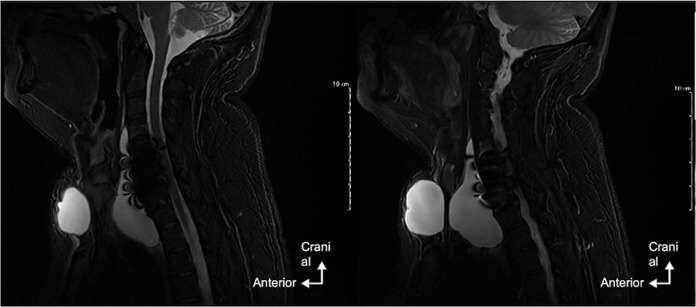
MRI in sagittal T2 section showing the anterior meningocele

**Figure 3:**
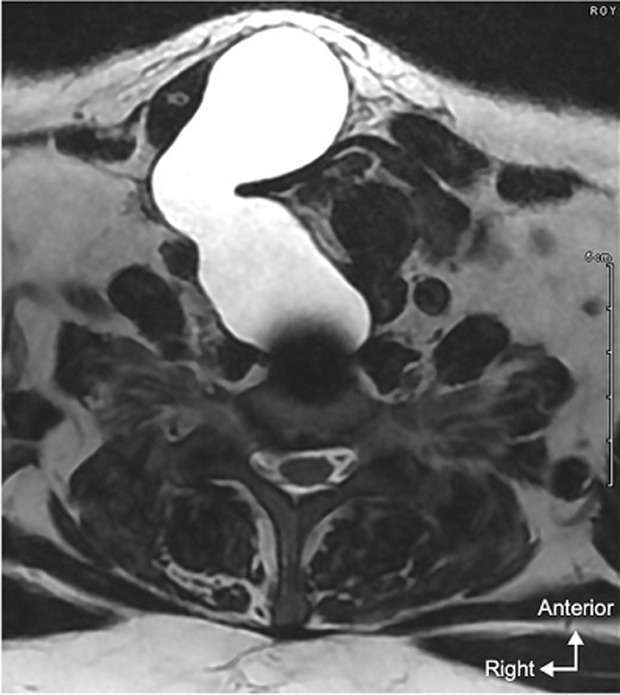
axial T2-weighted MRI showing anterior meningocele with left-displaced esophageal structures and skin fistula

**Diagnosis assessment:** a magnetic resonance imaging (MRI) was performed and a 10 x 7 x 4 cm pre-vertebral fluid collection was identified, with leftward discharge of the esophageal tract without any abnormality of the medullary cord. A suspicious hypersignal opposite the right foraminal C5-C6 may correspond to the intraoperative tear. Material-related artifacts did not allow accurate visualization of the dural disruption (Figure 2, Figure 3).

**Figure 4:**
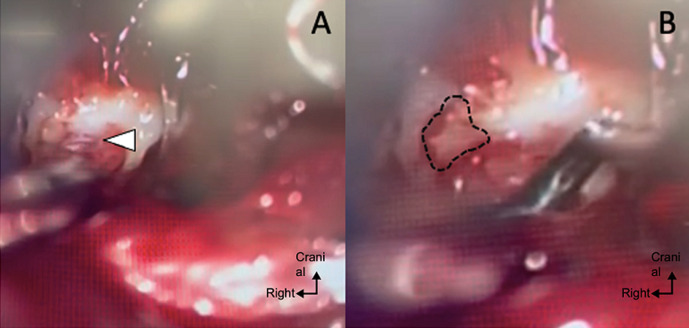
intraoperative microscopic photograph of the dural tear with steady pulsed flow of cerebrospinal fluid (A), and with placement of a fat patch in the breach prior to placement of the TachoSil (B)

**Therapeutic intervention:** the patient was reoperated on 27^th^ day of his initial surgery, under general anesthesia, in strict dorsal decubitus position, under C-arm fluoroscopy and high magnification microscope. First, we made a skin opening by repeating the incision from the first surgery. Sampling of liquid with a “clear water” appearance (200 ml) for systematic bacteriological analysis as usual. The sternocleidomastoid approach is taken from the anterior aspect of the cervical spine between the aerodigestive axis and the jugulocarotid bundle, following the plane of the meningocele, which creates a space for detachment up to the prosthesis C5-C6. Placement of a Caspar and TSI retractor to obtain the best possible exposure. Easy removal of the C5-C6 prosthesis without bony complications (no osteointegration of the prosthesis). Under the microscope, we observe a dural breach lateralized on the right side at the level of the nerve emergence (Figure 4). No more TachoSil seen at that place. A regular pulsatile flow of CSF was observed through a tiny hole of a few millimeters. It was therefore decided to place fatty tissue at the level of the breach and then apply a new TachoSil patch to close the breach, which was difficult to suture given the limited space available (Figure 4). We waited four minutes (minimum effective time) with a wet compress to improve the integration of the TachoSil. A Valsalva maneuver was used to eliminate any residual leakage intraoperatively. Given the absence of leakage, it was decided to place a new total disc prosthesis (identical to the previous one). Closure in two planes without the use of a suction drain, as usual.

**Figure 5:**
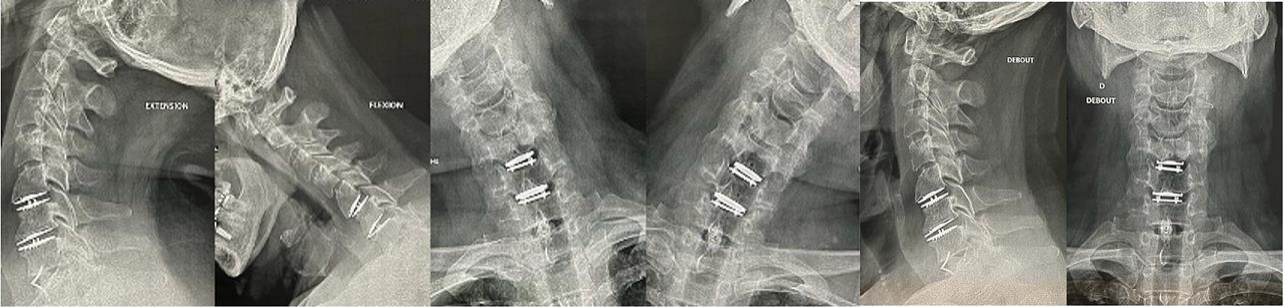
lateral (left) and frontal (right) static and dynamic X-ray of cervical spine showing the implants at 2 months

**Follow-up and outcomes:** following the operation, the patient did not have cervical immobilization. He was evaluated clinically (score JOA 17/17, VAS score 0/10) and radiologically at 2 and 6 months postoperatively and had no documented complications suggesting persistent leakage (Figure 5). In addition, tear and meningocele has not caused any complications of mechanical failure to the prosthesis for the moment (Figure 5).

**Patient perspective:** six months after the surgery, the patient was able to return to his sporting and professional activities without limitation. The patient is satisfied with the complete medical and surgical management.

**Informed consent:** the patient was informed about the case report, why his case was special and the authors´ intention to publish her case.

**Patient consent:** the patient has given her consent for his images and other clinical information to be reported in the journal.

## Discussion

We present the first case report of an anterior meningocele after a cervical arthroplasty. CSF leakage with neck mass effect after anterior discectomy is a rare event, but if not diagnosed and managed early it can have devastating consequences for the patient. The incidence of dural tears with anterior cerebrospinal fluid leak is 0.2 at 3% in the literature [1]. In addition, male gender, revision surgery, and surgery for ossification of the posterior longitudinal ligament are recognized risk factors for incidental durotomy [2]. Unfortunately, the management of this type of complication is not consensual and not well known by surgeons. Direct management and repair of the lesion is more difficult due to the limited working space and proximity to the spinal cord. The placement of an arthrodesis cage acts as a plug for most of the time to close the gap. In the case of arthroplasty, there is no buffering effect and if the tear is not repaired, an anterior meningocele may form.

Over time many techniques have been used to treat anterior dural tears; gelatin foam, fibrin glue, fascia, fat, muscle graft and sometimes postoperative placement of lumbar shunt (drain) [2,3-9]. In several cases and series in the literature, an external lumbar derivation (DLE) has been used with complete efficacy and resorption of the anterior meningocele. In our case, the presence of a punctiform disunion with liquid flow through the skin led us to reoperate the patient to close the active breach and to wash and change the prosthesis to limit the infectious risk [1,2,10-12].

Other teams, like ours, choose to perform a surgical revision to close the gap with similar results [13,14]. In 2019, the team of Gazzeri *et al*. presented a study of 8 cases treated with a TachoSil patch with very good efficacy [4]. TachoSil is a tissue sealant patch containing two layers, namely one collagenous layer, and a layer containing fibrinogen and human thrombin [15,16]. The sponge is manufactured from horse tendons. TachoSil reacts upon contact with blood, other body fluids or saline to form a clot that glues it to the tissue surface. Sealing is reached in a few minutes, and the sponge is absorbed by the body within several weeks [14]. The use of TachoSil was a very satisfactory option for us, allowing us to avoid enlarging the bone window to perform a direct suture. The treatment of anterior meningocele is not consensual in the literature and can vary from a simple external lumbar drainage to a more or less early surgical revision with closure of the dural tear by different techniques (direct suture, tissue graft, TachoSil...). A consensus on the management seems to be essential to help surgeons in their practices in front of this rare but difficult complication.

## Conclusion

Anterior dural rupture is a rare complication that can have serious consequences for patients. We present here the first case of dural tear in total disc arthroplasty. The TachoSil patch repair technique is reliable, reproducible and easy to use, and should be part of the therapeutic arsenal of spine surgeons. With the increasing number of anterior cervical discectomy procedures, a consensus is required on the management of post-discectomy anterior cerebrospinal fluid leakage to allow the spine surgeon to best manage this serious complication.

## References

[1] Yee TJ, Swong K, Park P (2020). Complications of anterior cervical spine surgery: a systematic review of the literature. Journal of Spine Surgery.

[2] Mazur M, Jost GF, Schmidt MH, Bisson EF (2011). Management of cerebrospinal fluid leaks after anterior decompression for ossification of the posterior longitudinal ligament: a review of the literature. Neurosurg Focus.

[3] Fountas KN, Kapsalaki EZ, Nikolakakos LG, Smisson HF, Johnston KW, Grigorian AA (2007). Anterior Cervical Discectomy and Fusion Associated Complications. Spine.

[4] Gazzeri R, Galarza M, Callovini G (2021). Use of tissue sealant patch (TachoSil) in the management of cerebrospinal fluid leaks after anterior cervical spine discectomy and fusion. Br J Neurosurg.

[5] Black P (2002). Cerebrospinal fluid leaks following spinal surgery: use of fat grafts for prevention and repair: Technical note. J Neurosurg Spine.

[6] Ahn JY, Kim SH (2009). A new technique for dural suturing with fascia graft for cerebrospinal fluid leakage in transsphenoidal surgery. Neurosurgery.

[7] Shaffrey CI, Spotnitz WD, Shaffrey ME, Jane JA (1990). Neurosurgical applications of fibrin glue: augmentation of dural closure in 134 patients. Neurosurgery.

[8] Hyun S-J, Rhim S-C, Ra Y-S (2009). Repair of a cerebrospinal fluid fistula using a muscle pedicle flap: technical case report. Neurosurgery.

[9] Lei T, Shen Y, Wang L, Cao J, Ding W, Ma Q (2012). Cerebrospinal Fluid Leakage during Anterior Approach Cervical Spine Surgery for Severe Ossification of the Posterior Longitudinal Ligament: Prevention and Treatment. Orthop Surg.

[10] Colombo GL, Bettoni D, Di Matteo S, Grumi C, Molon C, Spinelli D (2014). Economic and outcomes consequences of TachoSil®: a systematic review. Vasc Health Risk Manag.

[11] Montano N, Pignotti F, Auricchio AM, Fernandez E, Olivi A, Papacci F (2019). Results of TachoSil® associated with fibrin glue as dural sealant in a series of patients with spinal intradural tumors surgery. Technical note with a review of the literature. J Clin Neurosci Off J Neurosurg Soc Australas.

[12] Elder BD, Theodros D, Sankey EW, Bydon M, Goodwin CR, Wolinsky JP (2016). Management of Cerebrospinal Fluid Leakage During Anterior Cervical Discectomy and Fusion and Its Effect on Spinal Fusion. World Neurosurg.

[13] Spennato P, Rapanà A, Sannino E, Iaccarino C, Tedeschi E, Massarelli I (2007). Retropharyngeal cerebrospinal fluid collection as a cause of postoperative dysphagia after anterior cervical discectomy. Surg Neurol.

[14] Dorai Z, Morgan H, Coimbra C (2003). Titanium cage reconstruction after cervical corpectomy. J Neurosurg.

[15] Schaberg MR, Altman JI, Shapshay SM, Woo P (2007). Cerebrospinal fluid leak after anterior cervical disc fusion: an unusual cause of dysphagia and neck mass. The Laryngoscope.

[16] Fountas KN, Kapsalaki EZ, Johnston KW (2005). Cerebrospinal fluid fistula secondary to dural tear in anterior cervical discectomy and fusion: case report. Spine.

